# Efficacy and safety of adjuvant low-dose apatinib combined with SOX regimen versus SOX regimen in patients with resectable locally advanced gastric cancer: a cohort study

**DOI:** 10.3389/fphar.2026.1670012

**Published:** 2026-03-23

**Authors:** Liang Wang, Chenming Feng, Yi Nan, Yanjun Xu, Chao Zhang, Weipeng Wu, Yafei Duan, Haodong Zhang, Qiang Zhao

**Affiliations:** Department of Gastrointestinal Surgery, Heping Hospital, Changzhi Medical College, Changzhi, China

**Keywords:** adjuvant therapy, apatinib, disease-free survival, gastric cancer, S‐1 and oxaliplatin

## Abstract

**Objective:**

Apatinib combined with S‐1 and oxaliplatin (SOX) synergistically improves the prognosis of advanced gastric cancer patients, but its application as an adjuvant regimen has rarely been reported. This study aimed to investigate the efficacy and safety of adjuvant low-dose apatinib combined with SOX versus SOX alone in patients with resectable locally advanced gastric cancer.

**Methods:**

This cohort study included 110 patients with locally advanced gastric cancer (pTNM stage III) who underwent radical surgery. Among these patients, 70 patients received adjuvant low-dose apatinib (250 mg/day) combined with SOX regimen (named apatinib + SOX group), and 40 patients received adjuvant SOX regimen alone (named SOX group). Disease-free survival (DFS) and adverse events were recorded.

**Results:**

DFS was longer in apatinib + SOX group versus SOX group (*P* = 0.004). Specifically, the median DFS was not reached, and the 1-year/2-year cumulating DFS rates reached 75.7%/61.4% in apatinib + SOX group, whereas the median DFS was 15.0 (95% confidence interval: 10.0–20.0) months, and the 1-year/2-year accumulating DFS rates were 57.5%/35.0% in SOX group. After adjustment by multivariate Cox regression, apatinib + SOX (versus SOX) was independently related to prolonged DFS (hazard ratio = 0.316, *P* < 0.001). Moreover, hypertension showed a higher tendency in apatinib + SOX group compared to SOX group (22.9% versus 10.0%, *P* = 0.093), but did not reach statistical significance. However, incidences of other adverse events were not different between the two groups including: nausea and vomiting (*P* = 1.000), fatigue (*P* = 0.709), leukopenia (*P* = 0.103), pain (*P* = 0.564), thrombocytopenia (*P* = 0.113), anorexia (*P* = 0.564), and anemia (*P* = 0.686).

**Conclusion:**

Low-dose apatinib combined with SOX may be a feasible adjuvant regimen in patients with resectable locally advanced gastric cancer.

## 1 Introduction

Gastric cancer is a malignant epithelial neoplasm originating from the stomach and is the fifth most common malignancy and the fourth leading cause of cancer-related death worldwide (Bray et al., 2024). For patients with resectable locally advanced gastric cancer, surgical resection followed by adjuvant therapy is recommended to prolong survival (Sundar et al., 2025; Kim et al., 2024). Currently, the combination of chemotherapeutic drugs is the cornerstone of adjuvant therapy, as emphasized by the guidelines (Wang FH. et al., 2023; Ajani et al., 2025; Shitara et al., 2024). Among the optional adjuvant chemotherapy regimens, the S‐1 and oxaliplatin (SOX) regimen is well established and commonly used and provides survival benefits and acceptable tolerance (Zhang et al., 2021; Park et al., 2021; Jiang et al., 2020). However, investigating more feasible adjuvant regimens, such as the addition of targeted therapies to the SOX regimen, which may further improve the clinical management of patients with resectable locally advanced gastric cancer, is necessary (Song et al., 2023; Guo et al., 2023).

Angiogenesis supplies oxygen, nutrients, and growth factors to tumor cells, playing crucial roles in facilitating tumor growth, invasion, and metastasis (Liu et al., 2023). Vascular endothelial growth factor (VEGF) and its receptor (VEGFR) are key drivers of this process (Lorenc et al., 2024). In detail, the binding of VEGF to VEGFR activates downstream signaling pathways that regulate endothelial cell functions such as proliferation, migration, and survival, thereby promoting angiogenesis (Shi et al., 2025; Patel et al., 2023; Ghalehbandi et al., 2023). Given this critical role, antiangiogenic therapies targeting the VEGF/VEGFR signaling pathway have emerged as promising therapeutic strategies for various cancers, including gastric cancer (Liu et al., 2023; Xu et al., 2023).

Apatinib is an oral antiangiogenic drug that suppresses tumor progression through its specific inhibition of VEGFR-2 (Li et al., 2022; Fathi et al., 2020). Some clinical studies have reported the benefits of apatinib combined with SOX in advanced gastric cancer patients (Wang HF. et al., 2023; Ye et al., 2021; Deng et al., 2023). Moreover, according to previous studies, low-dose apatinib maintained efficacy while reducing toxicity compared to high-dose apatinib in gastric cancer patients (Wang et al., 2020; W et al., 2022). However, the application of apatinib plus SOX as adjuvant regimen in gastric cancer is seldom reported. Inspiringly, in patients with other cancer types such as hepatocellular carcinoma, high-grade glioma, and osteosarcoma, adjuvant regimens involving apatinib combined with camrelizumab, concurrent chemoradiotherapy, or chemotherapy achieve a satisfied efficacy and acceptable tolerance (Xia et al., 2022; Cai et al., 2024; Wang J. et al., 2023), shedding the light on apatinib adjuvant administration. Based on the above information, this study hypothesized that a low-dose apatinib combined with SOX as an adjuvant therapy regimen might be beneficial for resectable locally advanced gastric cancer patients.

Therefore, this study aimed to compare the efficacy and safety of adjuvant low-dose apatinib combined with SOX compared to SOX alone in resectable locally advanced gastric cancer patients.

## 2 Methods

### 2.1 Patients

This cohort study included 110 patients with resectable locally advanced gastric cancer between April 2021 to August 2023, who received low-dose apatinib combined with SOX regimen or SOX regimen alone as adjuvant therapy following radical surgery. The inclusion criteria were as follows: a) pathologically diagnosed as gastric or gastroesophageal junction cancer; b) aged over 18 years old and not limited to sex; c) locally advanced stage defined as pathological tumor-node-metastasis (TNM) stage III; d) received low-dose apatinib combined with SOX or SOX alone as adjuvant regimen after radical surgery. The exclusion criteria were as follows: a) Eastern Cooperative Oncology Group Performance Status (ECOG PS) score was ≥2; b) with distant metastasis; c) severe organ dysfunction in the brain, liver, kidney, or heart. Approval was obtained from the Ethics Committee of Heping Hospital, affiliated with Changzhi Medical College, with the approval number of 20,210,273. Informed consent was obtained from the patients or their families.

### 2.2 Treatment regimen

Patients received adjuvant low-dose apatinib combined with SOX regimen (apatinit + SOX group) or SOX regimen alone (SOX group) according to the clinical needs and patients’ willingness, which was not intervened. The adjuvant therapy was conducted about 4 weeks after radical surgery. The recommended regimen was six cycles with a 21-day cycle. Low-dose apatinib 250 mg was administered orally once a day. The SOX regimen was as follows: a) On Day 1: Intravenous infusion of oxaliplatin 130 mg/m^2^ + oral administration of tegafur (S-1) 40–60 mg/m^2^ twice (each time after breakfast and dinner). b) On Days 2–14: Oral administration of S-1 40–60 mg/m^2^ twice (each time after breakfast and dinner). c) On Days 15–21: Rest period (no medication). The treatment was discontinued if the patient developed intolerable toxicity, disease recurrence/progression, or a completion of six cycles of treatment.

### 2.3 Data collection and assessment

Baseline characteristics, including age, sex, body mass index (BMI), ECOG PS score, lesion location, pathological type, tumor differentiation, pathological TNM stage, human epidermal growth factor receptor 2 (HER2) -positivity, carbohydrate antigen 19–9 (CA199), and carcinoembryonic antigen (CEA), were collected. Patients were followed up to a maximum of 24 months or event occurrence (recurrence, death or lost follow up). During the follow up, routine imaging examinations were performed, and disease-free survival (DFS) was calculated. In addition, information on adverse events (AEs) was collected, which was evaluated according to CTCAE 5.0. The assessment was not blinded.

### 2.4 Statistical analysis

SPSS ver.26.0 (IBM, United States) was applied to data analyses. Minimum sample size was calculated as follows: with an assumption of 2-year accumulating DFS rate of 70% in apatinib + SOX group and 35% in SOX group, setting α as 0.05 and β as 0.20, and dropout rate of 10%, the minimum sample size of each group was 35 cases. The number of patients in each group exceeded the minimum sample size. The Student’s t-test, Chi-square test, Fisher’s exact test, and Mann-Whitney U test were applied to comparison analyses. The Kaplan-Meier curve was applied to show the accumulating DFS rate. The Log-rank test was used to compare the accumulating DFS rates between different groups. An enter-method multivariate Cox regression model was constructed to explore factors related to DFS. A *P* value < 0.05 indicated significance.

## 3 Results

### 3.1 Comparison of baseline characteristics between the two groups

In the apatinib + SOX group, the patients had a mean age of 62.8 ± 7.1 years, comprising 11 (15.7%) females and 59 (84.3%) males. Among these patients, 46 (65.7%) had an ECOG PS score of 0, and 24 (34.3%) had an ECOG PS score of 1. With respect to lesion location, 24 (34.3%) patients had gastric cancer, and 46 (65.7%) patients had gastroesophageal junction cancer. The tumor size was 4.8 ± 1.6 cm. In terms of the SOX group, the mean age of patients was 62.4 ± 6.4 years, and there were 9 (22.5%) females and 31 (77.5%) males. A total of 29 (72.5%) patients had an ECOG PS score of 0, and 11 (27.5%) patients had an ECOG PS score of 1. In addition, 18 (45.0%) patients had lesions located in the stomach, and 22 (55.0%) patients had lesions in the gastroesophageal junction. The tumor size was 5.1 ± 1.8 cm. Patients in both groups had pathological TNM stage III disease. After comparative analysis, the baseline characteristics, including demographic characteristics, ECOG PS score, tumor features, HER2 status, CA199 levels, and CEA levels (all *P* > 0.05), were well balanced between the two groups (Table 1).

**TABLE 1:** Clinical characteristics.

Items	Apatinib + SOX (N = 70)	SOX (N = 40)	*P* value
Age (years), mean ± SD	62.8 ± 7.1	62.4 ± 6.4	0.753
Sex, n (%)	​	​	0.375
Female	11 (15.7)	9 (22.5)	​
Male	59 (84.3)	31 (77.5)	​
BMI (kg/m^2^), mean ± SD	21.9 ± 2.8	23.7 ± 7.6	0.072
ECOG PS score, n (%)	​	​	0.462
0	46 (65.7)	29 (72.5)	​
1	24 (34.3)	11 (27.5)	​
Lesion location, n (%)	​	​	0.266
Gastric	24 (34.3)	18 (45.0)	​
Gastroesophageal junction	46 (65.7)	22 (55.0)	​
Pathological type, n (%)	​	​	1.000
Adenocarcinoma	65 (92.9)	37 (92.5)	​
Squamous cell carcinoma	5 (7.1)	3 (7.5)	​
Tumor differentiation, n (%)	​	​	0.698
Well-to-moderately differentiated	3 (4.3)	2 (5.0)	​
Moderately differentiated	12 (17.1)	9 (22.5)	​
Moderately-to-poorly differentiated	28 (40.0)	10 (25.0)	​
Poorly or undifferentiated	26 (37.1)	19 (47.5)	​
Unable to assess	1 (1.4)	0 (0.0)	​
Tumor size (cm), mean ± SD	4.8 ± 1.6	5.1 ± 1.8	0.437
Pathological TNM stage, n (%)	​	​	​
III	70 (100.0)	40 (100.0)	(−)
HER2-positive, n (%)	​	​	0.987
No	53 (62.4)	26 (65.0)	​
Yes	28 (32.9)	14 (35.0)	​
Undetected	4 (4.7)	0 (0.0)	​
CA199 (U/mL), mean ± SD	47.2 ± 93.9	45.4 ± 72.1	0.914
CEA (ng/mL), mean ± SD	19.4 ± 20.5	13.4 ± 21.8	0.155

SOX, S-1 and oxaliplatin; SD, standard deviation; BMI, body mass index; ECOG PS, eastern cooperative oncology group performance status; TNM, tumor-node-metastasis; HER2, human epidermal growth factor receptor 2; CA199, carbohydrate antigen 19–9; CEA, carcinoembryonic antigen.

### 3.2 Comparison of DFS between the two groups

**FIGURE 1:**
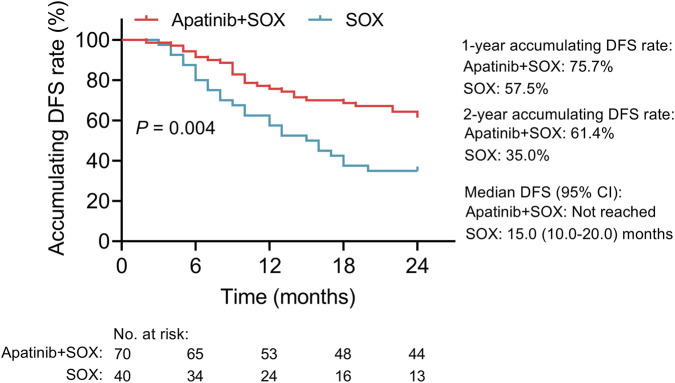
Comparison of DFS. Comparative analysis on DFS between resectable locally advanced gastric cancer patients receiving adjuvant low-dose apatinib combined with SOX and those receiving adjuvant SOX alone.

In the apatinib + SOX group, the median DFS [95% confidence interval (CI)] was not reached. The 1-year and 2-year accumulating DFS rates were 75.7% and 61.4%, respectively. For the SOX group, the median DFS (95% CI) was 15.0 (10.0–20.0) months; meanwhile, the 1-year and 2-year accumulating DFS rates were 57.5% and 35.0%, respectively. Specifically, the DFS was longer in the apatinib + SOX group than in the SOX group (*P* = 0.004) (Figure 1).

### 3.3 Multivariate cox regression analysis for DFS

To exclude the influence of potential confounding factors on the comparative analysis, multivariable Cox regression analysis was performed for adjustment. The multivariate analysis suggested that treatment (apatinib + SOX vs. SOX) was independently related to prolonged DFS [hazard ratio (HR) = 0.316, 95% CI: 0.166–0.602; *P* < 0.001]. Moreover, HER2 positivity (yes vs. no) (HR = 2.981, 95% CI: 1.596–5.569; *P* < 0.001) and CEA (per ng/mL) (HR = 1.020, 95% CI: 1.004–1.035; *P* = 0.012) independently predicted poor DFS. Other factors, such as age, sex, BMI, ECOG PS score, lesion location, pathological type, tumor differentiation, and CA199, were not independently linked with DFS (all *P* > 0.05) (Table 2).

**TABLE 2:** Enter-method multivariable Cox regression model on DFS.

Factors	*P* value	HR	95% CI	
			Lower limit	Upper limit
Treatment (Apatinib + SOX vs. SOX)	<0.001	0.316	0.166	0.602
Age (per year)	0.837	0.995	0.948	1.044
Sex (male vs. female)	0.293	0.675	0.324	1.404
BMI (per kg/m^2^)	0.216	0.928	0.824	1.045
ECOG PS score (1 vs. 0)	0.832	1.081	0.527	2.218
Lesion location (gastroesophageal junction vs. gastric)	0.976	0.991	0.532	1.846
Pathological type (squamous cell carcinoma vs. adenocarcinoma)	0.380	1.580	0.569	4.388
Poor tumor differentiation				
Well-to-moderately differentiated (reference)	(−)	1.000	(−)	(−)
Moderately differentiated vs. reference	0.966	1.032	0.234	4.564
Moderately-to-poorly differentiated vs. reference	0.934	1.060	0.269	4.180
Poorly or undifferentiated vs. reference	0.820	1.167	0.310	4.395
HER2-positive (yes vs. no)	<0.001	2.981	1.596	5.569
CA199 (per U/mL)	0.204	0.997	0.991	1.002
CEA (per ng/mL)	0.012	1.020	1.004	1.035

DFS, disease-free survival; HR, hazard ratio; CI, confidence interval; SOX, S-1 and oxaliplatin; BMI, body mass index; ECOG PS, eastern cooperative oncology group performance status; TNM, tumor-node-metastasis; HER2, human epidermal growth factor receptor 2; CA199, carbohydrate antigen 19–9; CEA, carcinoembryonic antigen.

### 3.4 Subgroup analyses for DFS

Subgroup analyses for DFS were performed based on the stratification of HER2 status and pTNM stage. DFS was prolonged in the apatinib + SOX group compared with the SOX group among HER2-positive patients (*P* = 0.032) (Supplementary Figure S1A), and showed a tendency to be longer in the apatinib + SOX group compared with the SOX group among HER2-negative patients, but did not reach statistical significance (*P* = 0.060) (Supplementary Figure S1B). Moreover, DFS was prolonged in the apatinib + SOX group compared to the SOX group among pTNM stage IIIA patients (*P* = 0.006) (Supplementary Figure S1C), was not different between the two groups among pTNM stage IIIB patients (*P* = 0.340) (Supplementary Figure S1D), and exhibited a tendency to be better in the apatinib + SOX group compared with the SOX group among pTNM stage IIIC patients, while did not reach statistical significance (*P* = 0.053) (Supplementary Figure S1E).

### 3.5 Comparison of the incidences of AEs between the two groups

The incidence of hypertension tended to be higher in the apatinib + SOX group than in the SOX group, while there was no statistical significance (22.9% vs. 10.0%, *P* = 0.093); particularly, the incidence of grade III/IV hypertension was 8.6% in apatinib + SOX group vs. 2.5% in SOX group (Table 3). For patients who occurred grade I/II hypertension, a close observation was performed; for patients who occurred grade III/IV hypertension, antihypertensive drug was used such as nifedipine.

**TABLE 3:** AEs.

AEs, n (%)	Apatinib + SOX			SOX			*P* value
	Any grade	Grade I/II	Grade III/IV	Any grade	Grade I/II	Grade III/IV	
Nausea and vomiting	28 (40.0)	24 (34.3)	4 (5.7)	16 (40.0)	13 (32.5)	3 (7.5)	1.000
Hypertension	16 (22.9)	10 (14.3)	6 (8.6)	4 (10.0)	3 (7.5)	1 (2.5)	0.093
Fatigue	12 (17.2)	9 (12.9)	3 (4.3)	8 (20.0)	7 (17.5)	1 (2.5)	0.709
Leukopenia	4 (5.7)	3 (4.3)	1 (1.4)	6 (15.0)	5 (12.5)	1 (2.5)	0.103
Pain	2 (2.9)	2 (2.9)	0 (0.0)	2 (5.0)	2 (5.0)	0 (0.0)	0.564
Thrombocytopenia	2 (2.9)	0 (0.0)	2 (2.9)	4 (10.0)	3 (7.5)	1 (2.5)	0.113
Anorexia	2 (2.9)	2 (2.9)	0 (0.0)	2 (5.0)	2 (5.0)	0 (0.0)	0.564
Anemia	1 (1.4)	1 (1.4)	0 (0.0)	1 (2.5)	1 (2.5)	0 (0.0)	0.686

AEs, adverse events; SOX, S-1 and oxaliplatin. *P* value was calculated by comparing AEs, of any grade.

No difference was observed in the incidences of nausea and vomiting (40.0% vs. 40.0%, *P* = 1.000), fatigue (17.2% vs. 20.0%, *P* = 0.709), leukopenia (5.7% vs. 15.0%, *P* = 0.103), pain (2.9% vs. 5.0%, *P* = 0.564), thrombocytopenia (2.9% vs. 10.0%, *P* = 0.113), anorexia (2.9% vs. 5.0%, *P* = 0.564), or anemia (1.4% vs. 2.5%, *P* = 0.686) between the two groups. Additionally, in both groups, most AEs were of grade I/II, and the incidences of AEs of grade III/IV were low (Table 3). Moreover, 7 (10.0%) patients in the apatinib + SOX group and 5 (12.5%) patients in the SOX group had drug dose reductions or delays due to AEs.

## 4 Discussion

Apatinib selectively competes with the ATP-binding site of the VEGFR2 receptor, which inhibits angiogenesis through blocking proangiogenic signaling pathways, thus suppressing the proliferation and migration of gastric cancer cells (Roviello et al., 2016; Jia et al., 2019). Moreover, apatinib induces tumor vessel normalization, increasing the delivery efficiency of chemotherapeutic drugs (Guo et al., 2024). Additionally, apatinib is considered to increase the sensitivity of gastric cancer to chemotherapeutic drugs (Xu et al., 2019). On the basis of the above evidence (Roviello et al., 2016; Jia et al., 2019; Guo et al., 2024; Xu et al., 2019), it is hypothesized that apatinib not only has direct antitumor activity but also may exert a synergistic effect with chemotherapeutic drugs in the treatment of gastric cancer.

Previous studies have shown that apatinib combined with SOX has favorable efficacy in advanced gastric cancer patients (Wang HF. et al., 2023; Ye et al., 2021; Deng et al., 2023; Kong et al., 2023; Peng et al., 2024). For example, a study revealed that apatinib combined with SOX resulted in a better treatment response and longer survival than did SOX alone in advanced gastric cancer patients (Wang HF. et al., 2023). Another study revealed that apatinib combined with SOX improved progression-free survival and overall survival compared with SOX alone in advanced gastric cancer patients (Deng et al., 2023). This study revealed that adjuvant low-dose apatinib combined with SOX prolonged DFS compared with SOX alone in patients with resectable locally advanced gastric cancer, which was similar to the findings of previous studies investigating the efficacy of apatinib combined with SOX in patients with advanced gastric cancer (Wang HF. et al., 2023; Ye et al., 2021; Deng et al., 2023). This result might be attributed to the following reasons (Bray et al., 2024): Apatinib inhibits the progression of gastric cancer through multiple methods, such as inhibiting the VEGF/VEGFR signaling pathway, suppressing the phosphatidylinositol 3-kinase/protein kinase B signaling pathway, and reducing the multidrug resistance of gastric cancer cells (Roviello et al., 2016; Jia et al., 2019; Zhao et al., 2021). (Sundar et al., 2025) Apatinib normalizes tumor blood vessels, which promotes the penetration of chemotherapeutic drugs (Guo et al., 2024). (Kim et al., 2024) Apatinib might synergize with SOX to improve antitumor efficacy (Deng et al., 2023).

Regarding adjuvant apatinib application in gastric cancer, the direct evidence is scare, and three literature are partially relevant (Hu J. et al., 2024; Zhan et al., 2025; Chang et al., 2025). One case report revealed a good efficacy of neoadjuvant and adjuvant SOX, apatinib, and camrelizumab in a patient with resectable locally advanced gastric cancer (Hu J. et al., 2024). Another cohort study disclosed that neoadjuvant immunotherapy, anti-angiogenic therapy (specifically apatinib), and chemotherapy (SOX), and adjuvant chemotherapy realized a higher response rate and satisfied survival in patients with locally advanced gastric cancer (Zhan et al., 2025). Moreover, a protocol of single-arm phase II trial investigating the efficacy and safety of neoadjuvant SOX, apatinib, and camrelizumab followed by adjuvant SOX and camrelizumab in patients with locally advanced gastric or gastroesophageal junction adenocarcinoma was recently issued but the results were not reported (Chang et al., 2025).

Notably, potential differences in the baseline characteristics of patients might have affected the results of the comparison analysis. Thus, to exclude the influence of confounding factors, this study used an enter-method multivariate Cox regression model for adjustment. The adjusted findings revealed that apatinib + SOX was independently superior to SOX alone in prolonging DFS in patients with resectable locally advanced gastric cancer. Moreover, owing to the heterogeneity of gastric cancer, the outcomes of patients after treatment might be quite different (Zubarayev et al., 2019; Rocken, 2023). Thus, exploring the associations of clinical characteristics with DFS may provide more information for predicting the prognosis of these patients. This study suggested that HER2-positive status and elevated CEA were independently related to shortened DFS in patients with resectable locally advanced gastric cancer. The corresponding explanations are as follows (Bray et al., 2024): HER2-positive status represents a heightened malignant phenotype characterized by enhanced proliferative capacity and aggressive behaviors, ultimately reflecting shortened DFS (Hu HH. et al., 2024; Cheng, 2024). (Sundar et al., 2025) Elevated CEA is associated with enhanced invasive and metastatic potential, indicating a greater tumor burden (Zhu et al., 2024).

Moreover, the safety profile of apatinib should be taken into consideration even at a low dose. Previous studies have suggested that the most common AEs of apatinib include hypertension, vomiting, proteinuria, leucopenia, granulocytopenia, and fatigue in gastric cancer patients (Ma et al., 2025; Han et al., 2025). In this study, the most frequent AEs of patients with resectable locally advanced gastric cancer who received adjuvant low-dose apatinib combined with SOX included nausea and vomiting, hypertension, and fatigue, which were similar to the findings of previous studies (Ma et al., 2025; Han et al., 2025). This study also revealed that, in addition to a slightly greater incidence of hypertension, adjuvant low-dose apatinib combined with SOX did not increase the incidence of other adverse events (AEs) than SOX alone did. Additionally, all AEs were predominantly grade I/II and were mild and manageable. Overall, the results indicated that the addition of low-dose apatinib to SOX as an adjuvant regimen resulted in an acceptable safety profile.

Several limitations should be noted (Bray et al., 2024): This study was a single-center study, which might lead to selection bias to some extent and less generalizability. Thus, the efficacy and safety of adjuvant low-dose apatinib combined with SOX should be further verified in larger-scale, multicenter studies (Sundar et al., 2025). The follow-up period of this study was relatively short. Future studies with longer follow-up periods are needed for further verification (Kim et al., 2024). The observational design of this study might have caused some confounding factors, and randomized controlled trials are needed for verification (Wang FH. et al., 2023). The OS data were not analyzed in this study, since the relevant data were not completed. This condition was due to two aspects: on one hand, the study design focused on DFS evaluation that “Patients were followed up to a maximum of 24 months or event occurrence (recurrence, death or lost follow up).”, when patients occurred recurrence, the endpoint reached, and the OS data based on this timepoint were not accurate; on another hand, after recurrence, the majority of patients received subsequent treatments in internal medicine departments such as Oncology Department or Gastroenterology Department instead of our department (Gastrointestinal Surgery Department), or in other hospitals, their subsequent data were hard to access.

In conclusion, adjuvant low-dose apatinib combined with SOX achieves a longer DFS than SOX alone with a comparable safety profile in resectable locally advanced gastric cancer patients, implying its potency to be a feasible adjuvant regimen in these patients.

## Data Availability

The original contributions presented in the study are included in the article/Supplementary Material, further inquiries can be directed to the corresponding author.

## Supplementary material

SUPPLEMENTARY FIGURE S1 Subgroup analyses. Comparative analysis on DFS between the apatinib + SOX group and the SOX group in patients with HER2-positive **(A)**, HER2-negative **(B)**, pTNM stage IIIA **(C)**, pTNM stage IIIB **(D)**, and pTNM stage IIIC **(E)**.
