# Supplementary figures and images for: Efficacy and safety of adjuvant low-dose apatinib combined with SOX regimen versus SOX regimen in patients with resectable locally advanced gastric cancer: a cohort study
Source: Front Pharmacol. 2026 Mar 23;17:1670012. doi: 10.3389/fphar.2026.1670012 (PMC13051217; doi:10.3389/fphar.2026.1670012)

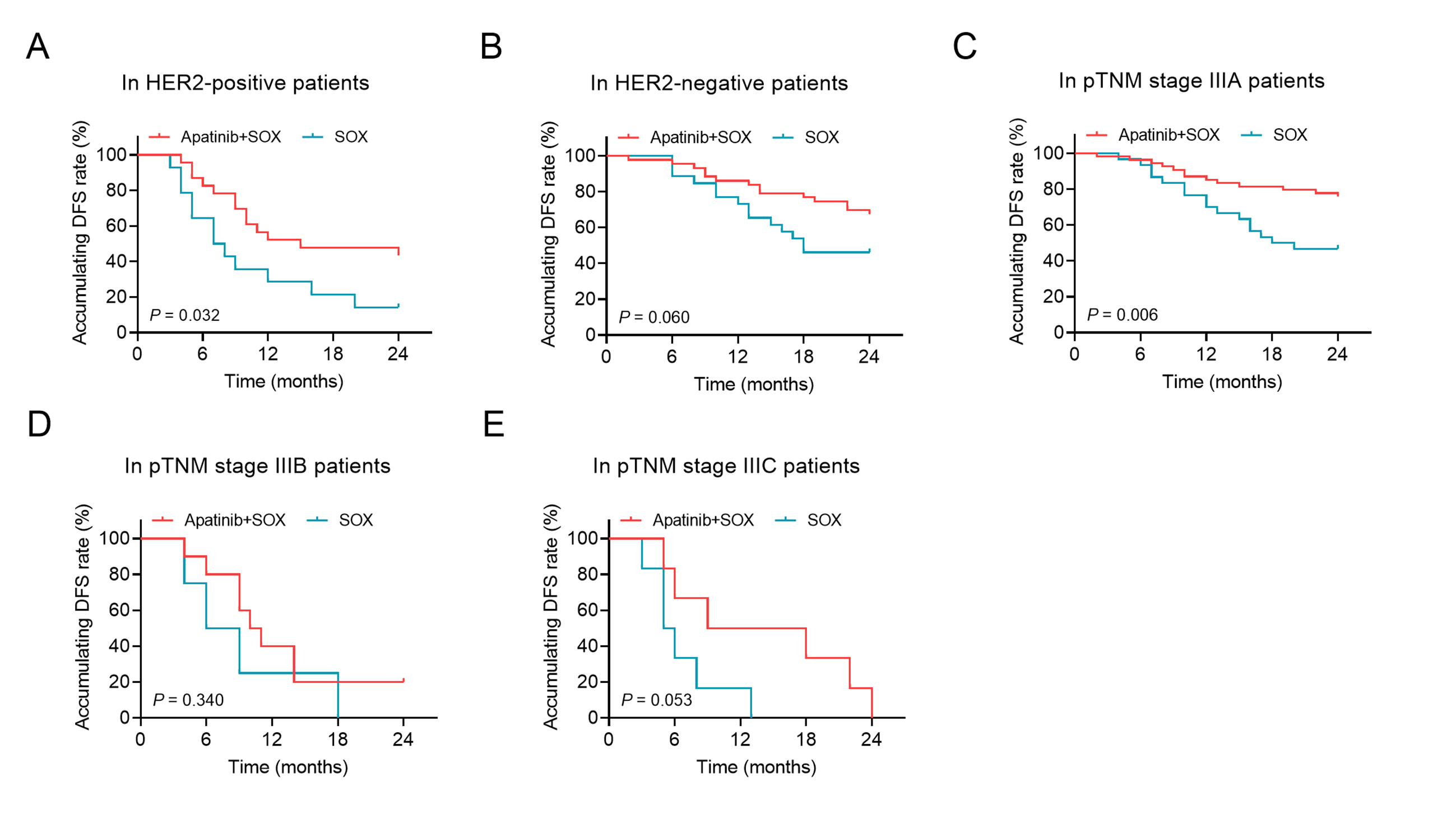

Supplement: Supplementary file 1 [file Image1.tif]
